# Isolation of Allosteric Tryptase Inhibitor from Methanol Extract of Rhubarb and Enhancement of Its Tryptase Inhibitory Activity by Compounds That Were Screened by In Silico Screening

**DOI:** 10.3390/molecules30061341

**Published:** 2025-03-17

**Authors:** Hidetoshi Fujii, Moeno Ito, Kentaro Nishioka, Katsutoshi Nishino, Takanao Otsuka, Kazuhiro Irie, Takashi Tanaka, Masaya Nagao

**Affiliations:** 1 Graduate School of Biostudies, Kyoto University, Kyoto 606-8502, Japanito.moeno.57r@st.kyoto-u.ac.jp (M.I.);; 2 Department of Applied Chemistry and Biotechnology, Okayama University of Science, Okayama 700-0005, Japan; 3 Graduate School of Agriculture, Kyoto University, Kyoto 606-8502, Japan; irie.kazuhiro.2z@kyoto-u.jp; 4 Graduate School of Biomedical Sciences, Nagasaki University, Nagasaki 852-8521, Japan; t-tanaka@nagasaki-u.ac.jp

**Keywords:** tryptase, allosteric inhibitor, rhubarb, tannin, in silico screening

## Abstract

Tryptase, which is abundant in human mast cells and is involved in allergic inflammations such as asthma, is a serine protease. We isolated a tryptase inhibitor, procyanidin B8 3,3′-di-*O*-gallate (PB8GG’), a tannin, from the methanol extract of rhubarb (RHEI RHIDOMA), which is a traditional Chinese medicine (Kampo medicine in Japan). Since it did not inhibit another serine protease trypsin, PB8GG’ specifically inhibited tryptase. A standard kinetic analysis of the inhibitory fashion of PB8GG’ against tryptase suggested that PB8GG’ inhibited tryptase in an allosteric manner. We searched for other tannins like PB8GG’ expected to bind tryptase using AutoDock vina. Two ellagitannins, carpinins B and E, isolated from young leaves of *Carpinus japonica* were selected as candidates of tryptase inhibitors. Carpinins B and E themselves had almost no inhibitory activity against tryptase but enhanced the inhibitory activity of PB8GG’ against tryptase. This is an example that shows that a combination of an allosteric inhibitor with other compounds that bind but have no inhibitory activity can be used to develop a clinically useful combinatorial enzyme inhibitor.

## 1. Introduction

Tryptase is a serine protease, like trypsin, and is secreted from mast cells involved in allergic inflammations. Human tryptase has more than three known isoforms, among which α- and β-tryptases are especially important. β-tryptase is predominant in lung mast cells [1] and is released from granules of mast cells as a proteolytically active tetramer complexed with heparin [2].

Tryptase has various biological functions in allergic diseases, such as asthma and rheumatoid arthritis, suggesting that tryptase is a good target of clinical medicine [3], but to our knowledge, a clinical medication targeting tryptase has yet to be developed. A synthetic serine protease inhibitor nafamostat mesilate (FUT-175) developed as an anticoagulant is clinically administered for the treatment of acute pancreatitis [4,5]. Potent tryptase inhibition by nafamostat mesilate has been reported [6], but this compound has not been clinically administered as a tryptase-targeting medicine.

A clinical trial of APC-366, another tryptase inhibitor, for asthma was abandoned as some patients treated with APC-366 developed bronchospasms [7]. An anti-tryptase antibody that dissociates the active tetramer into an inactive monomer was developed for asthma associated with increased mast cell tryptase [8], but the clinical availability of this antibody remains unknown [9].

In this study, after screening plant extracts for tryptase inhibitory activity in various samples including extracts from north African plants, we found tryptase inhibitory activity in the methanol extract of roots and rhizomes of rhubarb, a traditional Japanese (Kampo) or Chinese medicine. Rhubarb is known to contain anthraquinons, such as aloe-emodin, emodin, rhein, chrysophanol, and physcion [10], and bianthrones, such as sennoside *a* [11], and has cathartic, psychotropic, analgesic, anti-inflammatory, and anti-bacterial effects.

We found that a kind of tannin derived from rhubarb can specifically inhibit tryptase in this study, prompting us to search for other tannins with tryptase inhibitor activity by an approach in silico using AutoDock vina [12,13] and we found two ellagitannins, carpinins B and E [14], to be candidates of allosteric tryptase inhibitors. Although these ellagitannins had little inhibitory activity against tryptase, these tannins enhanced the tryptase inhibitory activity of the tannin we identified in this study. In our previous study, we found that a combination of two allosteric inhibitors is useful for designing a stronger mixture for the inhibition of cathepsin K [15]. The results of this study will encourage the development of a specific enhancer of an allosteric inhibitor that can bind to the allosteric site of the enzyme using the approach in silico, irrespective of its inhibitory activity.

## 2. Results

### 2.1. The Identification of a Tryptase Inhibitor in the Extract of Rhubarb (RHEI RHIZOMA) and an Analysis of Specificity of the Tryptase Inhibitor

#### 2.1.1. The Purification of a Tryptase Inhibitor from the Methanol Extract of Rhubarb

**Figure 1:**
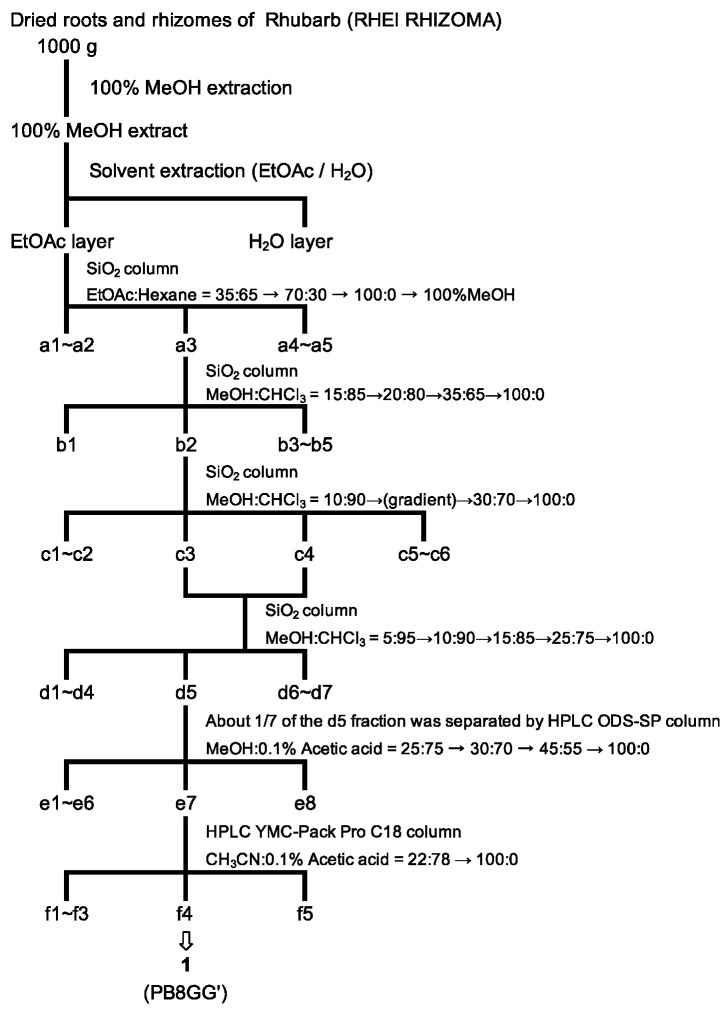
Schematic diagrams of the purification of a tryptase inhibitor from the dried roots and rhizomes of rhubarb. Compound **1** (PB8GG’) is isolated in the f4 fraction.

The procedure for the purification of a tryptase inhibitor from the roots and rhizomes of rhubarb is shown in Figure 1.

#### 2.1.2. The Identification of the Tryptase Inhibitor in the Methanol Extract of Rhubarb

**Figure 2:**
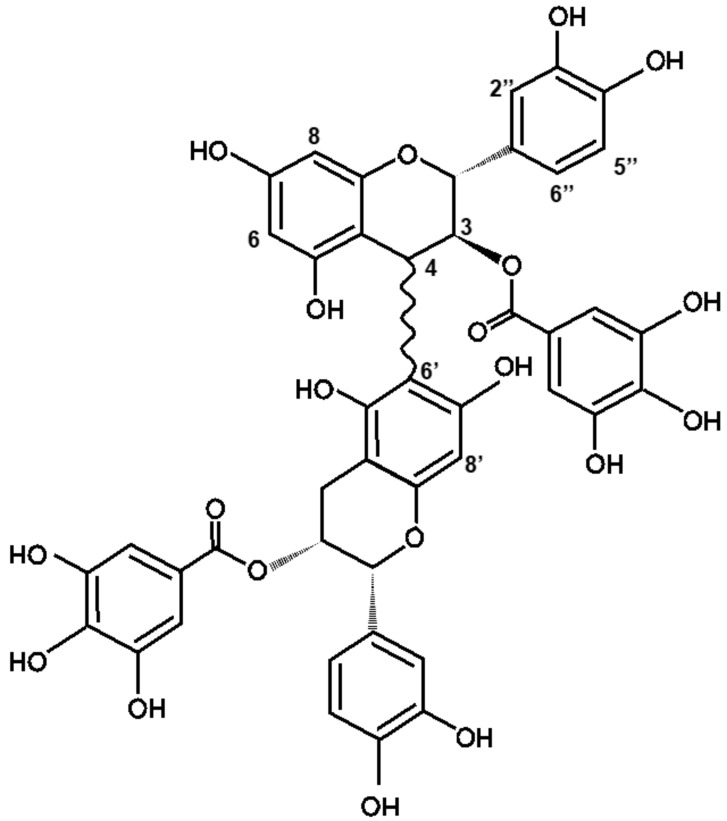
The chemical structure of PB8GG’, an inhibitor of tryptase isolated from the extract of rhubarb. The absolute configuration of carbon 4 of PB8GG’ has not yet been specified (drawn in a wavy line).

Compound **1**, an orange-colored solid, was isolated as a tryptase inhibitor isolated at the f4 fraction from the methanol extract of rhubarb (Figure 1). The molecular formula was C_44_H_34_O_20_ because the HR-ESI-MS spectrum exhibited an [M + H]^+^ ion peak at *m*/*z* 883.1679 (calcd. for C_44_H_35_O_20_: *m*/*z* 883.1722). Also, *m*/*z* 441.0797 was detected as a fragment ion, indicating that compound **1** was a dimer with a partial structure of *m*/*z* 441 (Figure S1). In addition, a fragment ion at *m*/*z* 729.1414 was detected in the HR-ESI-MS/MS analysis in negative mode with a precursor ion at *m*/*z* 881.15 (Figure S2). This result suggested that compound **1** has a substructure of *m*/*z* 152, which is known to be a gallate group. Therefore, the structure of compound **1** was considered to be a dimer of the ester of catechin or epicatechin and gallic acids, i.e., a compound of procyanidins with two molecules of gallic acid attached. Therefore, the ^1^H-NMR spectrum of compound **1** was compared with that of the procyanidin B2 3,3′-di-*O*-gallate standard (Santa Cruz), which has the structure of epicatechin gallate-(4→8′)-epicatechin gallate. The chemical shifts of H-2 (δ = 5.29 ppm) and H-3 (δ = 5.23 ppm) of compound **1** were high-shifted compared with those of procyanidin B2 3,3′-di-*O*-gallate (H-2 5.54 ppm, H-3 5.40 ppm, respectively). This showed that the upper side was catechin not epicatechin (Figure S3). Moreover, the peak at H-8′ (δ = 6.10 ppm) observed in compound **1** was not detected in the procyanidin B2 3,3′-di-*O*-gallate standard (Figure S3). The structures of the procyanidins are composed of catechin groups binding to carbons at the 4→8′ or 4→6′ position. This indicated that compound **1** was composed of procyanidin B8, which had the structure of (+)-catechin and (−)-epicatechin bound at the 4→6′ position [16]. Furthermore, compound **1** has two ester-linked gallic acids at the 3 and 3′ positions, suggesting that compound **1** was procyanidin B8 3,3′-di-*O*-gallate (PB8GG’). The absolute configuration of carbon 4 of PB8GG’ has not yet been identified (drawn in a wave line) (Figure 2).

### 2.2. Mode of Tryptase Inhibition by PB8GG’ and Determination of Kinetic Parameters

#### 2.2.1. Specific Inhibition of Tryptase by PB8GG’

**Figure 3:**
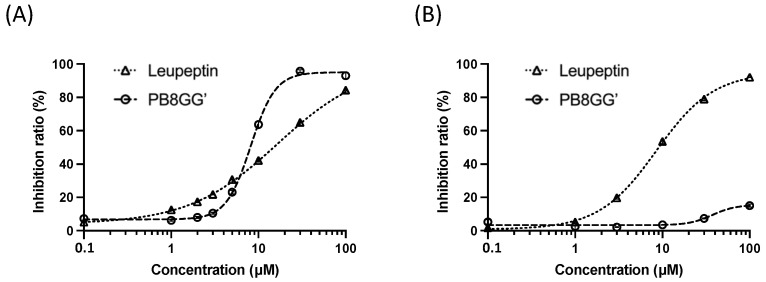
Dose-dependent inhibition of tryptase (**A**) or trypsin (**B**) by PB8GG’ (open circle, dashed line) or leupeptin (open triangle, dotted line). Results are presented as mean ± SE (*n* = 3). GraphPad prism 9.5.1 was used for curve fitting. In the calculation of curve fitting, maximal inhibition is set at less than 100%.

Tryptase is a kind of serine protease like trypsin. A known serine protease inhibitor, leupeptin, inhibited both tryptase and trypsin (IC_50_ = 14.9 ± 1.25 μM and 8.40 ± 0.31 μM, respectively), and PB8GG’ inhibited tryptase (IC_50_ = 7.78 ± 0.17 μM) but showed little inhibitory activity against trypsin (Figure 3A,B), suggesting that PB8GG’ is a tryptase specific inhibitor.

#### 2.2.2. Allosteric Inhibition of Tryptase by PB8GG’

**Figure 4:**
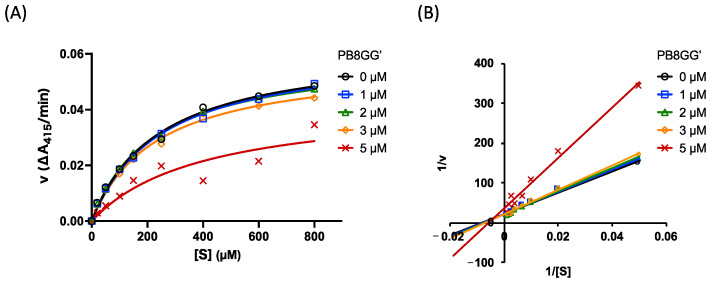
Inhibition of tryptase activity by PB8GG’. Eight concentrations of substrate (**A**,**B**) (20, 50, 100, 150, 250, 400, 600, 800 μM) and five concentrations of the inhibitor (**A**,**B**): 0 (open circle in black), 1 (open square in blue), 2 (open triangle in green), 3 (open diamond in orange), 5 (cross, in red) μM of PB8GG’. Lineweaver–Burk plots for the inhibition of tryptase by PB8GG’ (**B**) are used to determine the inhibition mode.

The inhibition mode and inhibition constant of PB8GG’ were determined in the presence of various concentrations of PB8GG’ (Figure 4). According to the Lineweaver–Burk plot, the mode of inhibition of PB8GG’ seemed to be mixed-type non-competitive (Figure 4B). The inhibition constant calculated for PB8GG’ was 5.02 ± 0.99 μM (*K*_i_^app^). PB8GG’ was considered to serve as an allosteric inhibitor against tryptase.

### 2.3. In Silico Screening of the Other Tryptase Inhibitors Using AutoDock Vina

#### 2.3.1. Carpinins B and E Are Candidate Compounds That Bind to Tryptase

**Figure 5:**
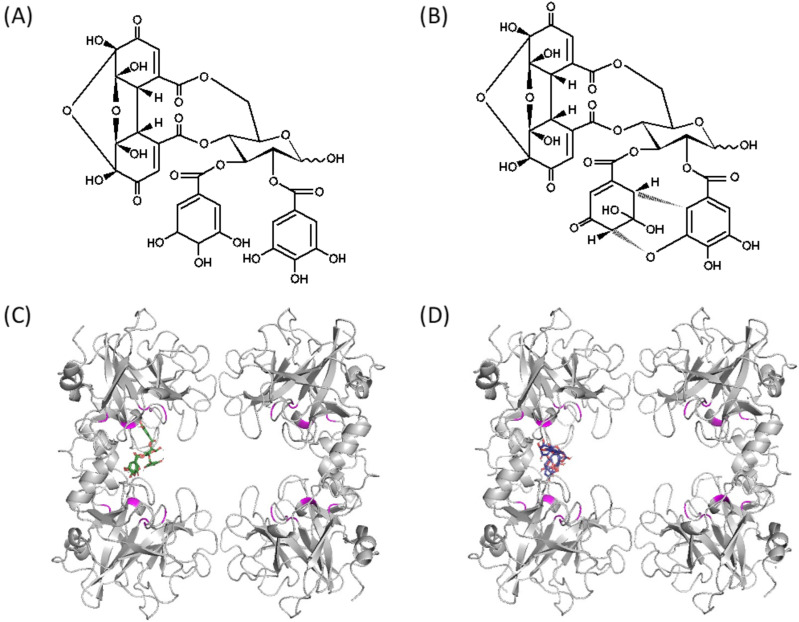
Structures of carpinin B (**A**) and carpinin E (**B**) and speculated binding sites of carpinin B (**C**) and carpinin E (**D**) to tryptase (PBD code 1A0L). The carbon backbones of carpinins B and E are described in green (**C**) and blue (**D**), respectively. The active center of each tryptase subunit of the tryptase tetramer is colored in magenta. Carpinin B and carpinin E that bind to symmetrical locations in the tetramer of tryptase are omitted (**C**,**D**). The predicted binding sites of carpinins B and E with the best docking score are shown (**C**,**D**).

Since PB8GG’ is a kind of tannin, we used AutoDock vina for the in silico screening of known ellagitannins to find other compounds that bind to tryptase. Carpinins B and E, which had the predicted binding sites with the best docking sore of −10.1 and −10.9 kcal/mol, respectively, were selected as the candidates (Figure 5A,B). The predicted binding sites of carpinins B and E with the best docking score to tryptase are shown (Figure 5C,D).

#### 2.3.2. Enhancement of the Tryptase Inhibitory Activity of PB8GG’ by Carpinins B and E

**Figure 6:**
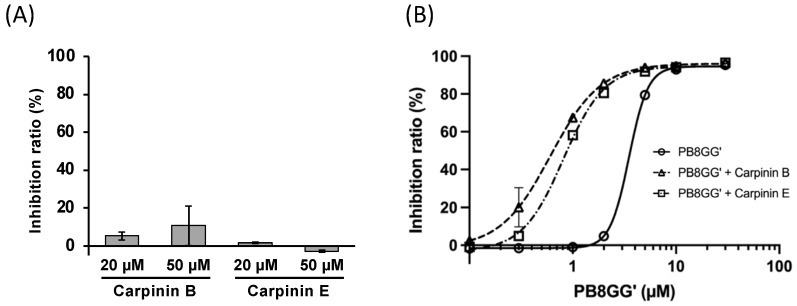
Almost little or no inhibitory activity of carpinins B and E to tryptase (**A**). Combination of PB8GG’ and carpinins B or E enhanced the inhibitory activity of PB8GG’ to tryptase (**B**). Results are presented as mean ± SE (*n* = 3).

Carpinins B and E alone had little inhibitory activity against tryptase at the dose of 20 and 50 μM (Figure 6A) but enhanced the inhibitory activity of PB8GG’ to tryptase at the dose of 50 μM (IC_50_ = 0.62 ± 0.054 μM and 0.84 ± 0.024 μM, respectively) (Figure 6B).

## 3. Discussion

Tryptase, especially β-tryptase secreted from lung mast cells, is involved in asthma. Since tryptase plays an important role in allergic inflammation and rheumatoid arthritis, tryptase could become a target of clinical medicine. However, a drug directly targeting tryptase has not been developed. MTPS9579A, an allosteric antibody to tryptase, was developed [8], but it had little effect on asthma patients in a phase 2 study [9]. Imatinib, a KIT inhibitor that inhibits tryptase release from mast cells, is attracting attention in the field of clinical medicine for severe asthma but remains to be developed [17]. Tryptase inhibitors, such as APC-366 [18] and nafamostat mesilate (FUT-175) [6,19,20], have not been developed as clinical medication for tryptase inhibition. The clinical application of APC-366 was abandoned because it caused bronchospasms in the treated patients [7,21]. Nafamostat mesilate is utilized as an anti-coagulant [22].

In this study, we found an allosteric tryptase inhibitor from the extract of rhubarb “RHEI RHIZOMA” a typical Japanese (Kampo) or Chinese medicine whose extracts contain anti-SARS coronavirus 3C-like protease activity [23]. Tryptase composed of four subunits with heparin is a kind of serine protease [24,25]. We found that PB8GG’ derived from rhubarb specifically inhibited tryptase but not trypsin, another serine protease; in contrast, leupeptin inhibited both tryptase and trypsin (Figure 3A,B). Studies are currently underway to determine the binding site of PB8GG’ by molecular dynamics simulation and by producing a recombinant human β-tryptase by *Komagataella phaffii* (synoym *Pichia pastoris*) [26] with or without mutations in the predicted binding sites. Since PB8GG’ drastically inhibited tryptase in the dose range of 1 to 10 μM (Figure 3A), we speculate that PB8GG’ binds to multiple sites of the tryptase tetramer: the binding of PB8GG’ to the last location of the multiple binding sites of tryptase may trigger inhibition. In future research, we will determine the binding site of PB8GG’ by comparing the inhibitory activity against the wild-type and mutant tryptase. PB8GG’ at the dose of 1–3 μM very weakly inhibited tryptase activity and at 5 μM clearly inhibited tryptase activity (Figure 3A). A kinetic analysis of the inhibition of tryptase by PB8GG’ by a Lineweaver–Burk plot reflected this dose-dependent inhibitory potential of PB8GG’ (Figure 4B) and showed the non-competitive, strictly speaking, mixed-type non-competitive inhibition of tryptase by PB8GG’. Taking this together the fact that PB8GG’ did not inhibit trypsin, another type of serine protease (Figure 3B), PB8GG’, is supposed to bind to the tryptase allosterically and not to the active center of tryptase.

Since PB8GG’ is a kind of tannin, we screened candidates of tryptase inhibitors among the commercially available ellagitannins using AutoDock vina and focused on two compounds, carpinins B and E derived from *Carpinus japonica* [14] (Figure 5A,B). Carpinins B and E had almost no inhibitory activity at the dose of 50 μM (Figure 6A); however, both carpinins B and E at 50 μM significantly enhanced the inhibitory activity of PB8GG’ to tryptase (Figure 6B). To explain the enhancement of the inhibitory activity of PB8GG’ against tryptase by carpinin B and carpinin E, we speculate that carpinin B and carpinin E may make PB8GG’ bind easier to the binding sites of tryptase or the binding of PB8GG’ to tryptase may make carpinin B and carpinin E bind to tryptase by the conformation change in tryptase and enhance the inhibitory activity of PB8GG’against tryptase. Our previous study showed that a combination of two independent allosteric inhibitors that were supposed to bind different allosteric sites, pheophytin *a* or pheophorbide *b* and NSC13345 or NSC94914, additively inhibited cathepsin K [15]. In this study, carpinins B and E that were speculated to bind to tryptase using AutoDock vina in silico had little inhibitory activity against tryptase (Figure 6A) but enhanced the inhibitory activity of PB8GG’ against tryptase (Figure 6B), suggesting that in silico screening for inhibitors of enzymes is a useful tool for finding new drugs irrespective of their inhibitory activity against the enzymes. Recently, allosteric inhibitors, such as asciminib, an inhibitor of BCR-ABL1 tyrosine kinase [27], and deucravacitinib, an inhibitor of tyrosine kinase 2 (TYK2) [28], approved as clinical medicine are receiving attention since allosteric inhibitors are generally expected to exhibit high specificity with minor side effects [29]. In recent drug screening, AlphaFold 2 and very recently AlphaFold 3 have become available for predicting protein structures. Molecular dynamics simulation plays an important role for in silico drug screening, and artificial intelligence (AI) accelerates this process [30]. An in silico study can provide many candidates of allosteric inhibitors that are supposed to bind to the target proteins like carpinins B and E in this study, but most candidates are ineffective with no inhibitory activity. This is because it is difficult to speculate the movement of the target protein after the binding of such inhibitor candidates, whether the inhibitor candidates block the binding of the substrate or disturb the movement in the enzyme reaction. In this study, we found that carpinins B and E, which have little inhibitory activity against tryptase, enhance the inhibitory activity of PB8GG’ to tryptase (Figure 6B). Many compounds with little inhibitory activity against the target proteins whose binding sites are expected to be candidates of allosteric inhibition by the in silico technique may be useful as “enhancers” of other known allosteric inhibitors among inactive compounds for the production of new drugs. In our previous study, we constructed a new PPARγ agonist created by the covalent bonding of two compounds, GW9662, an antagonist of PPARγ, and a compound that exhibits PPARγ agonist activity in the presence of GW9662 only [31]. The production of a new hybrid inhibitor by the covalent binding of two allosteric inhibitors or an inhibitor and an “enhancer” may enable the creation of a new drug with stronger enzyme inhibitory activity.

## 4. Materials and Methods

### 4.1. Chemical Reagents and Instruments

Organic solvents for fractionation were purchased from Nacalai Tesque, Kyoto, Japan. Triton-X100 was purchased from Fujifilm Wako Pure Chemical, Osaka, Japan. Optical rotation was measured on a P-2200 (JASCO, Tokyo, Japan). ^1^H and ^13^C NMR spectra were measured and recorded on Avance III 400 and 500 (Burker, Bremen, Germany). High-resolution ESI-MS data were obtained on a Waters ACQUITYTM UPLC with the Xevo G2-S QTof mass spectrometer (Nihon Waters, Shinagawa, Japan).

### 4.2. Preparation of Extract of Rhubarb and Purification of Tryptase Inhibitors

The dried roots and rhizomes of rhubarb, RHEI RHIZOMA (Daiou (TAKEDA), Tochimototenkaido Co., Ltd., Osaka, Japan) (1.0 kg), were extracted with methanol (5.0 L) for one week at room temperature. After filtration, filtrates were evaporated until dry in vacuo at 40 °C to obtain methanol extracts (281.26 g). The purification steps of the extracts are described in Figure 1. The methanol extracts were partitioned between ethyl acetate and H_2_O. Silica gel column chromatography (Wako gel C-200, Fujifilm Wako Pure Chemical Corporation, Osaka, Japan; YFLC AI-580, Yamazen Corporation, Osaka, Japan) and HPLC (Chromaster, Hitachi High-Tech Science Corporation, Tokyo, Japan) using ODS-SP (GL Sciences, Osaka, Japan) and YMC Tiant C18 (YMC, Kyoto, Japan) were employed for further purification, as shown in Figure 1.

### 4.3. Procyanidin B8 3,3′-Di-O-Gallate (***1***)

${\left[\alpha \right]}_{D}^{16}$ + 13.5 (*c* 0.002, MeOH); ^1^H-NMR (400 MHz, CD_3_OD): δ (ppm) 6.98 (2H, *s*, galloyl H-2, 6), 6.96 (1H, *d*, *J* = 1.8 Hz, H-2″), 6.95 (2H, *s*, galloyl H-2′ and 6′), 6.85 (2H, *dd*, *J* = 1.8 and 8.1 Hz, H-6″ and H-6‴), 6.72 (1H, *s*, H-2‴), 6.69 (1H, *d*, *J* = 8.2 Hz, H-5‴), 6.66 (1H, *d*, *J* = 8.2 Hz, H-5″), 6.10 (1H, *s*, H-8′), 6.06 (1H, *d*, *J* = 2.3 Hz, H-8), 5.95 (1H, *d*, *J* = 2.3 Hz, H-6), 5.56 (1H, br *s*, H-3′), 5.29 (1H, br *s*, H-2), 5.23 (1H, br *s*, H-3), 5.06 (1H, *s*, H-2′), 4.56 (1H, *s*, H-4), 3.06 (1H, br *d*, *J* = 12.7 Hz, H-4′), 2.88 (1H, br *d*, *J* = 16.4 Hz, H-4′); ^13^C-NMR (125 MHz, CD_3_OD): δ (ppm) 168.5 (galloyl C-7 and 7′), 158.6 (C-8a or C-8a′), 156.7 (C-8a or C-8a′), 147.2 (galloyl C-4 and 4′), 146.91 (C-4″ or C-4‴), 132.3 (C-3″ or C-3‴), 132.2 (C-3″ or C-3‴), 120.3 (C-6‴), 120.2 (C-2‴), 117.0 (C-5‴), 116.9 (C-5″), 116.0 (C-2″), 115.9 (C-6″), 111.3 (galloyl C-2′ and 6′), 111.2 (galloyl C-2 and 6), 101.4 (C-8′), 97.7 (C-6), 96.8 (C-8), 79.5 (C-2′), 70.8 (C-3′), 36.0 (C-4), 28.2 (C-4′) (Figures S1–S7). HR-ESI-MS *m*/*z* [M + H]^+^ = 883.1679, (calcd for C_44_H_35_O_20_). 

### 4.4. Measurement of Inhibitory Activity of the Inhibitor Against Tryptase and Trypsin

Tryptase from a human lung was purchased from Sigma-Aldrich (St. Louis, MO, USA). The activity of tryptase was measured in 100 mM Tris-HCl buffer, pH 8.0, 100 mM glycerol, 0.02 mg/mL heparin sodium salt and 167 µM N-(*p*-Tosyl)-Gly-Pro-Lys 4-nitroanilide acetate salt (Sigma-Aldrich) as a substrate. Prior to addition of the substrate, the inhibitor was preincubated with the enzyme for 30 min at room temperature to allow the establishment of the enzyme–inhibitor complex. The substrate was then added, and after incubation for 30 min at 37 °C, the enzyme activity was measured from the increase in absorbance at 415 nm. The assays were performed using a 384-well microplate (CELLSTAR 384-well 781182, Greiner Bio-One, Kremsmünster, Austria) and absorbance was measured using a Powerscan 4 plate reader (DS pharma biomedical, Osaka, Japan). The inhibition ratio was calculated using the following equation:

$$\mathrm{inhibition}\;\mathrm{ratio}\;\left(\%\right)=\left(1-\frac{A\left(E+S+I\right)-A\left(S\right)-A\left(I\right)+A\left(\mathrm{blank}\right)}{A\left(E+S\right)-A\left(S\right)}\right)\times 100$$

A(x): absorbance intensity (415 nm); E: enzyme; S: substrate; I: inhibitor; blank: buffer.

Trypsin from a hog pancreas was purchased from Nacalai Tesque, Kyoto, Japan. The activity of trypsin was measured in 200 mM Tris-HCl buffer, pH 7.8, 20 mM CaCl_2_ and 385 µM Benzoyl-L-arginine p-nitroanilide monohydrochloride (Bz-L-Arg-*p*NA·HCl) (Peptide Institute, Osaka, Japan) as a substrate. Prior to addition of the substrate, the inhibitor was preincubated with the enzyme for 10 min at room temperature to allow the establishment of the enzyme–inhibitor complex. The substrate was then added, and after incubation for 60 min at 37 °C, the enzyme activity was measured from the increase in absorbance at 415 nm. The assays were performed using a 384-well microplate and absorbance was measured using a Powerscan 4 plate reader.

The inhibitory activity against tryptase and the trypsin of leupeptin and PB8GG’ were measured at 0.1–100 µM, along with that of ellagitannins (carpinin B or carpinin E) isolated by our group [14] at 20–50 µM. The inhibitory activity of a combination of various concentrations of PB8GG’ and 50 µM ellagitannins was also assayed.

Drawing curves of the inhibition and calculation of IC_50_ were performed by GraphPad Prism (Version 9.5.1).

*K*_i_^app^ was calculated using the Morrison’s formula with GraphPad Prism. The mean value of *K*_m_ from the kinetic analysis (described in next Section 4.5) was substituted as a constant in the formula.

### 4.5. Kinetic Analysis of Tryptase Inhibition

For kinetic studies of tryptase, inhibition assays were carried out as described in the preceding paragraph. The substrate concentrations were fixed at 0, 20, 50, 100, 150, 250, 400, 600, and 800 µM, while the PB8GG’ concentrations were 0, 1, 3, 5 µM. The kinetic parameters were calculated by curve fitting using GraphPad Prism.

### 4.6. Docking Study

PyMOL (Version 2.3.5) (https://pymol.org/2/, accessed on 4 March 2020), Avogadro (Version 1.2.0) (https://avogadro.cc/, accessed on 3 June 2016), AutoDock Tools (Version 1.5.6), and AutoDock vina (Version 1.1.2) (http://vina.scripps.edu/, accessed on 17 December 2020) were downloaded from the internet.

The protein structure file was downloaded from Protein Data Bank (Tryptase, PDB ID: 1A0L), and the ligand, water molecules, NO_3_ molecules, and amino acids constituting the leader sequence were removed using PyMOL. The compound files were created using MolView (https://molview.org, accessed on 11 March 2025) and converted to pdb format by Avogadro.

The docking study was performed using AutoDock vina without specifying the coupling pocket (blind docking). Finally, the lowest energy pose of the calculated results was selected and visualized with PyMOL.

## 5. Conclusions

PB8GG’, a tryptase inhibitor isolated from the methanol extract of a rhubarb, specifically inhibited tryptase in an allosteric manner. Carpinins B and E, candidates of trypsin binding compounds that were screened by in silico screening, themselves had almost no inhibitory activity against tryptase but enhanced the inhibitory activity of PB8GG’ against tryptase.

## Data Availability

The data that support the findings of this study are available from the corresponding author (M.N.) upon reasonable request.

## Supplementary Materials

The following supporting information can be downloaded at: https://www.mdpi.com/article/10.3390/molecules30061341/s1, Figure S1. HR-ESI-MS spectrum in positive mode of compound **1**. Figure S2. HR-ESI-MS/MS spectrum in negative mode of compound **1**. Figure S3. ^1^H NMR (400 MHz) spectrum of procyanidin B2 3,3′-di-*O*-gallate standard (a) and compound **1** (b) in CD_3_OD. Figure S4. ^13^C NMR (125 MHz) spectrum of compound **1** in CD_3_OD. Figure S5. HSQC spectrum of compound **1** in CD_3_OD. Figure S6. HMBC spectrum of compound **1** in CD_3_OD. Figure S7. ^1^H-^1^H COSY spectrum of compound **1** in CD_3_OD.
