# Supplementary material for: Isolation of Allosteric Tryptase Inhibitor from Methanol Extract of Rhubarb and Enhancement of Its Tryptase Inhibitory Activity by Compounds That Were Screened by In Silico Screening
Source: Molecules. 2025 Mar 17;30(6):1341. doi: 10.3390/molecules30061341 (PMC11944477; doi:10.3390/molecules30061341)
Supplement: Supplementary file 1 [file molecules-30-01341-s001.zip › molecules-3479742-supplementary.pdf]

Supplementary Materials

Section S1. MS and NMR spectra

Figure S1. HR-ESI-MS spectrum in positive mode of compound **1**.

Figure S2. HR-ESI-MS/MS spectrum in negative mode of compound **1**.

Figure S3. ^{1}H NMR (400 MHz) spectrum of procyanidin B2 3,3'-di-*O*-gallate standard (a) and compound **1** (b) in CD_3OD.

Figure S4. ^{13}C NMR (125 MHz) spectrum of compound **1** in CD_3OD.

Figure S5. HSQC spectrum of compound **1** in CD_3OD.

Figure S6. HMBC spectrum of compound **1** in CD_3OD.

Figure S7. ^{1}H-^{1}H COSY spectrum of compound **1** in CD_3OD.

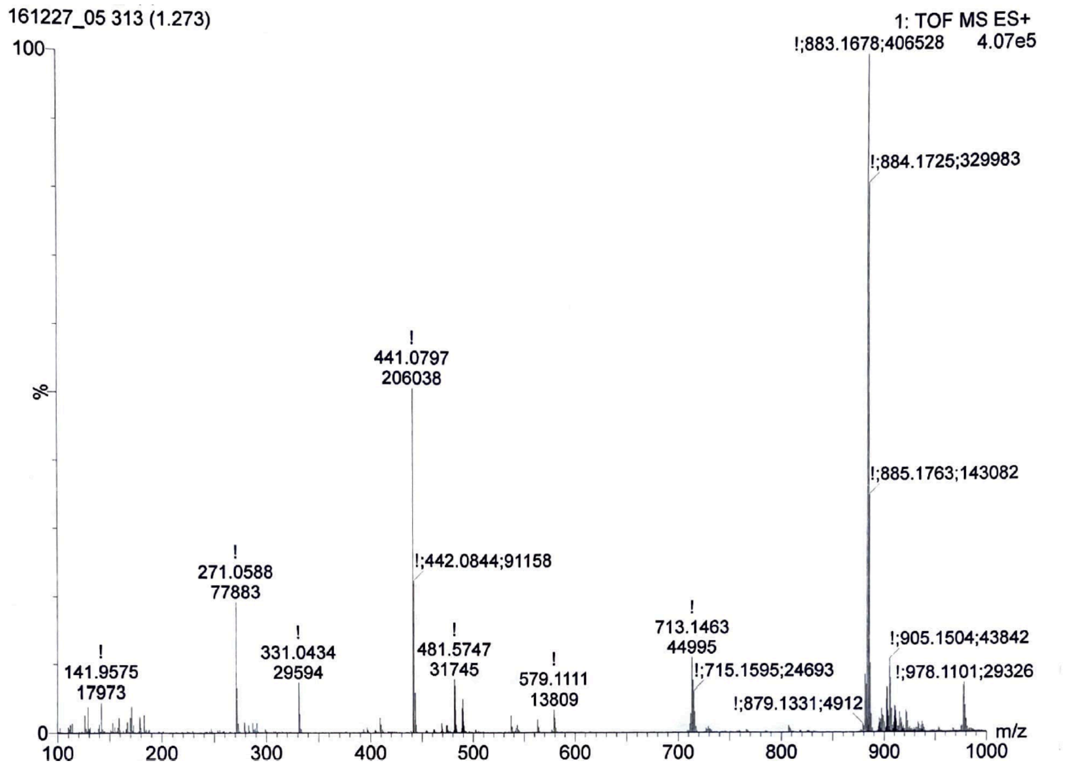

Figure S1. HR-ESI-MS spectrum in positive mode of compound **1**.

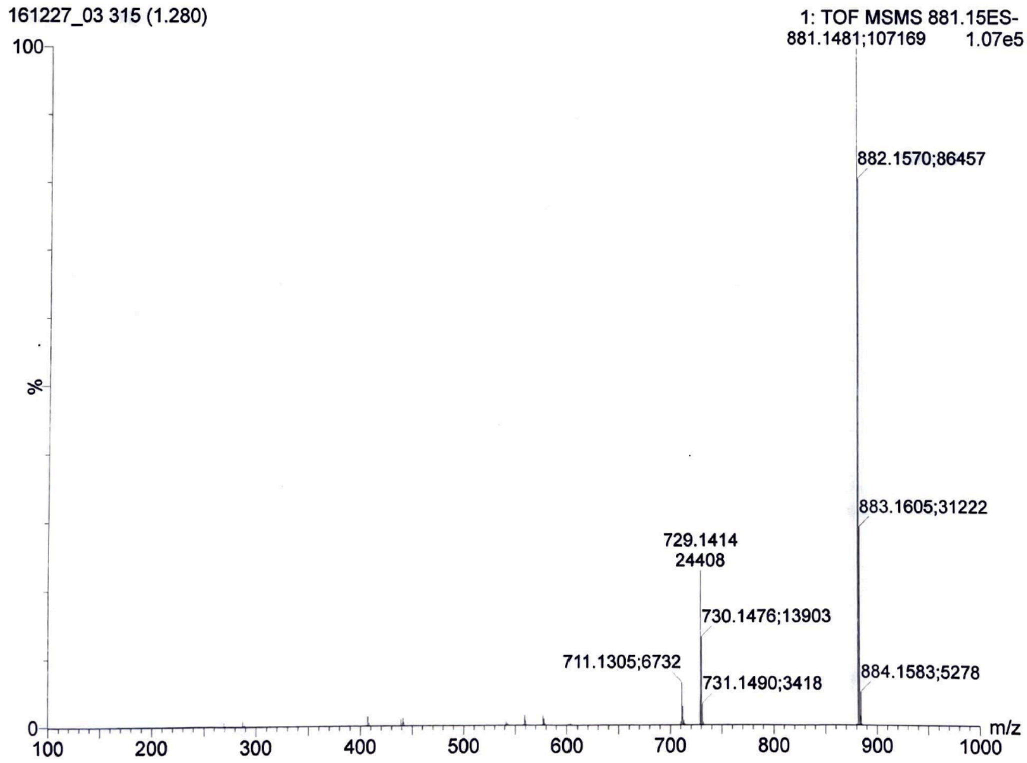

Figure S2. HR-ESI-MS/MS spectrum in negative mode of compound **1**.

(a)

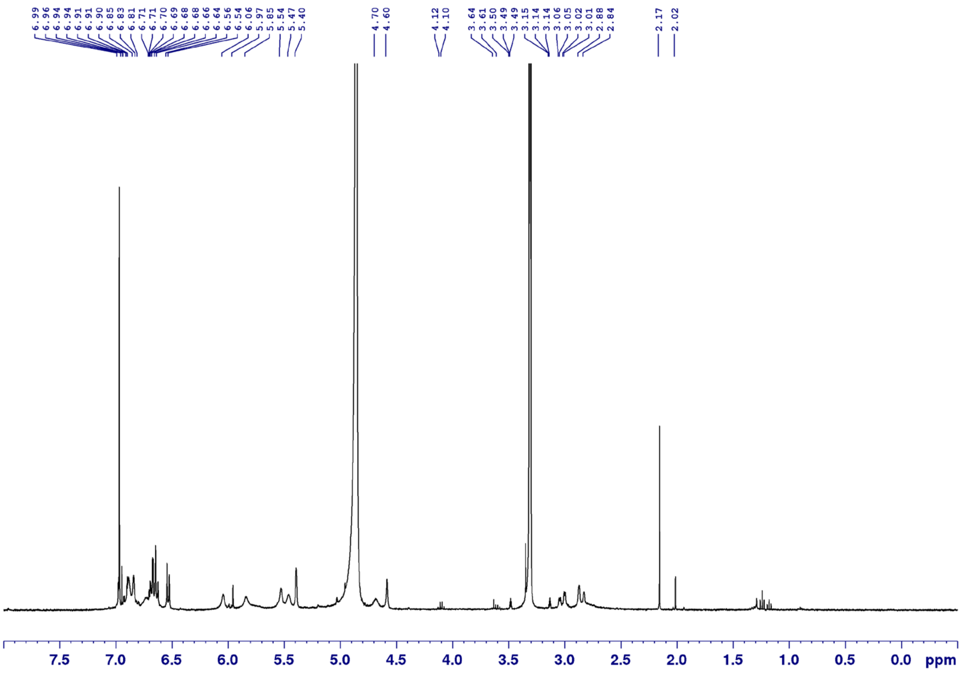

(b)

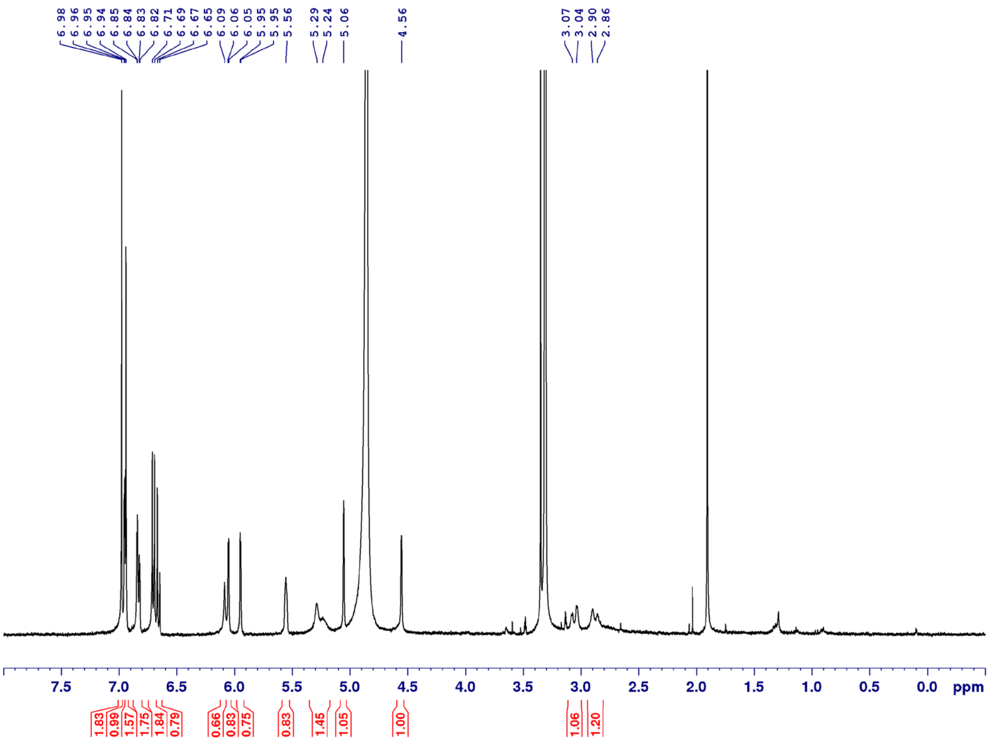

Figure S3. ^{1}H NMR (400 MHz) spectrum of procyanidin B2 3,3'-di-*O*-gallate standard (a) and compound **1** (b) in CD_3OD.

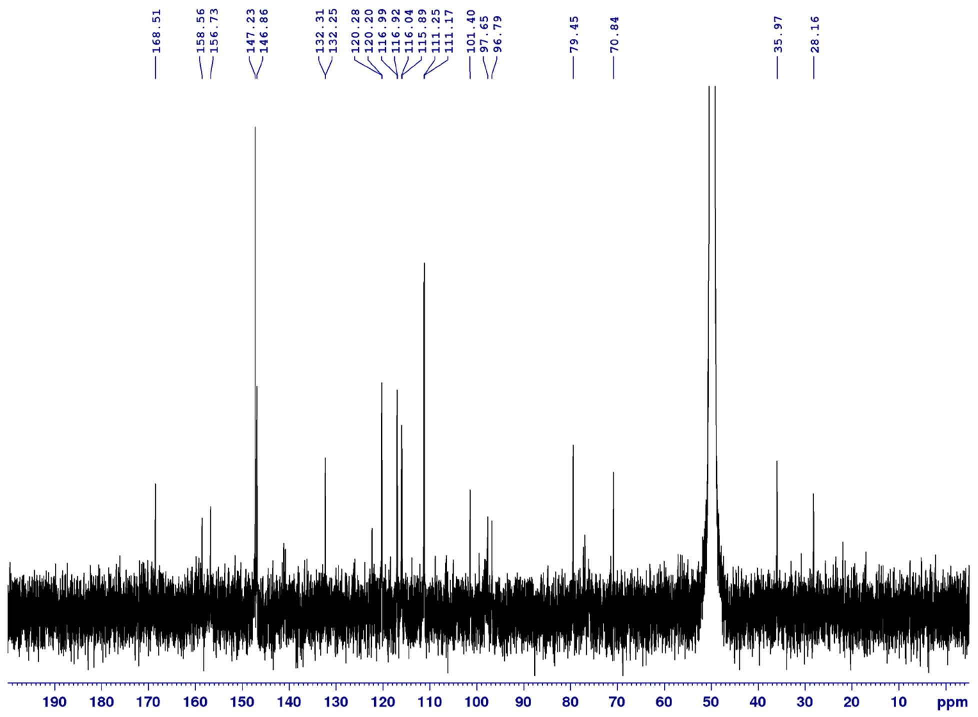

Figure S4. ^{13}C NMR (125 MHz) spectrum of compound **1** in CD_3OD.

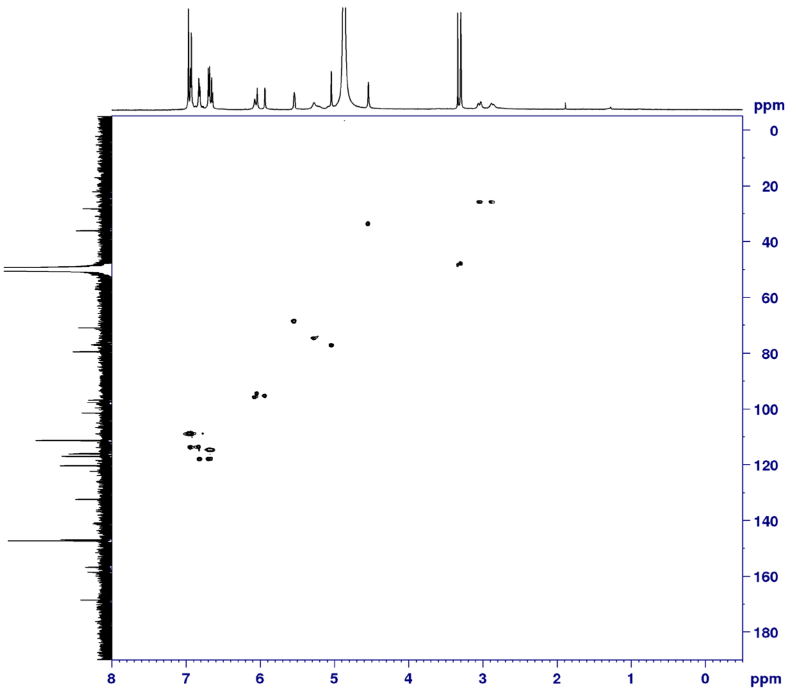

Figure S5. HSQC spectrum of compound **1** in CD_3OD.

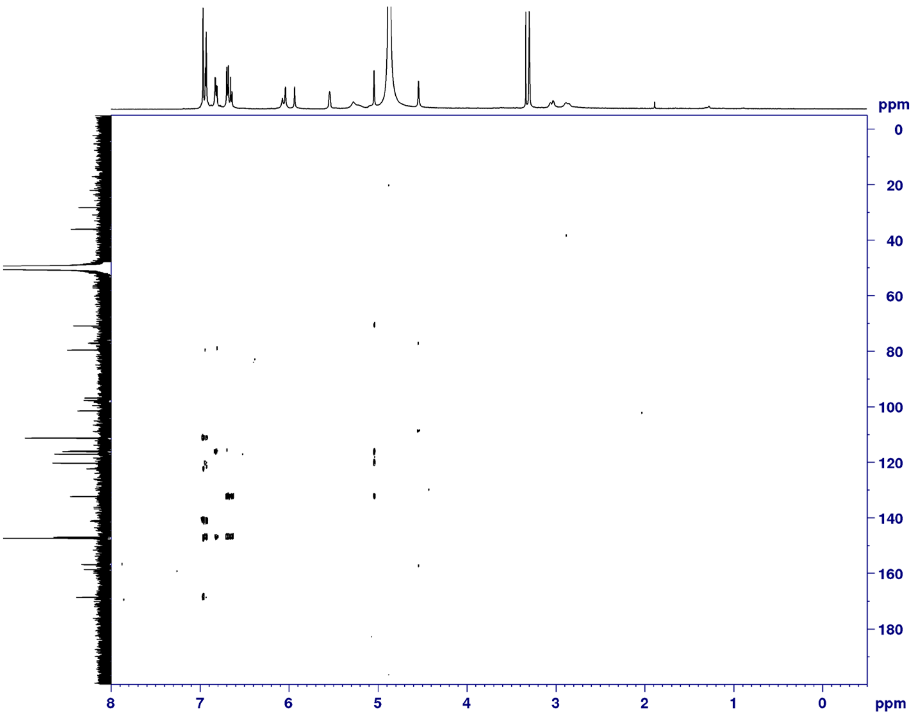

Figure S6. HMBC spectrum of compound **1** in CD_3OD.

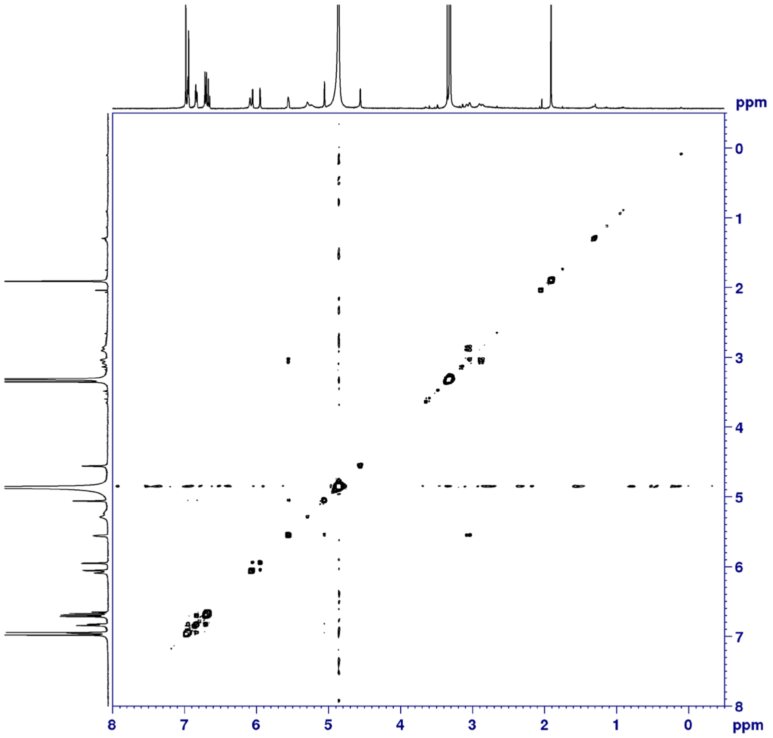

Figure S7. ^{1}H-^{1}H COSY spectrum of compound **1** in CD_3OD.
